# Differences in bioavailability and cognitive-enhancing activity exerted by different crystal polymorphs of latrepirdine (Dimebon^®^)

**DOI:** 10.3389/fphar.2023.1091858

**Published:** 2023-02-22

**Authors:** Boris I. Gorin, Elena A. Tukhovskaya, Alina M. Ismailova, Gulsara A. Slashcheva, Oksana A. Lenina, Konstantin A. Petrov, Ilya V. Kazeev, Arkady N. Murashev

**Affiliations:** ^1^ Bigespas Ltd, London, United Kingdom; ^2^ Biological Testing Laboratory, Branch of Shemyakin and Ovchinnikov Institute of Bioorganic Chemistry, Russian Academy of Sciences, Pushchino, Russia; ^3^ Arbuzov Institute of Organic and Physical Chemistry, Federal Research Center of Kazan Scientific Center, Russian Academy of Sciences, Kazan, Russia; ^4^ Federal State Budgetary Institution National Medical Research Center of Oncology Named After N.N. N.N. Blokhin» of the Ministry of Health of Russia, Moscow, Russia

**Keywords:** latrepirdine, polymorph, rat, pharmacokinetics, scopolamine, passive avoidance test

## Abstract

**Introduction:** Pharmacokinetic characteristics as well as cognitive-enhancing nootropic activity of latrepirdine (Dimebon^®^) in relationship with its polymorphic forms have been studied in SD and Wistar rats.

**Methods:** The pharmacokinetics of six polymorphs (A, B, C, D, E, F) of latrepirdine were studied in male SD rats after 7 days of oral administration in corn oil at a dose of 10 mg/kg once a day. Blood and brain samples were taken on the 7th day of administration at 15 min, 30 min, 60 min and 120 min after administration and analyzed for latrepirdine content by LC-MS. The cognitive-enhancing nootropic effect was studied in male and female Wistar rats after 9 days of oral administration in corn oil at a dose of 10 mg/kg, after prior administration of scopolamine, an agent that causes memory impairment similar to that in Alzheimer’s disease. The animals’ cognitive function was studied in the passive avoidance test.

**Results:** When studying the pharmacokinetics, the highest bioavailability both in the blood and in the brain was demonstrated by polymorph E, whose AUC was the highest relative to other polymorphs. In the study of the cognitive-enhancing nootropic effect, polymorph E also showed the highest activity, whose values of the latent period of entering the dark chamber did not differ from control animals, and differed from other polymorphs.

**Conclusion:** Thus, the crystal structure has been shown to play a key role in the bioavailability and efficacy of latrepirdine, and polymorph E has also been shown to be a promising drug for the treatment of neurodegenerative diseases associated with memory impairment, such as Alzheimer’s disease.

## 1 Introduction

**FIGURE 1:**
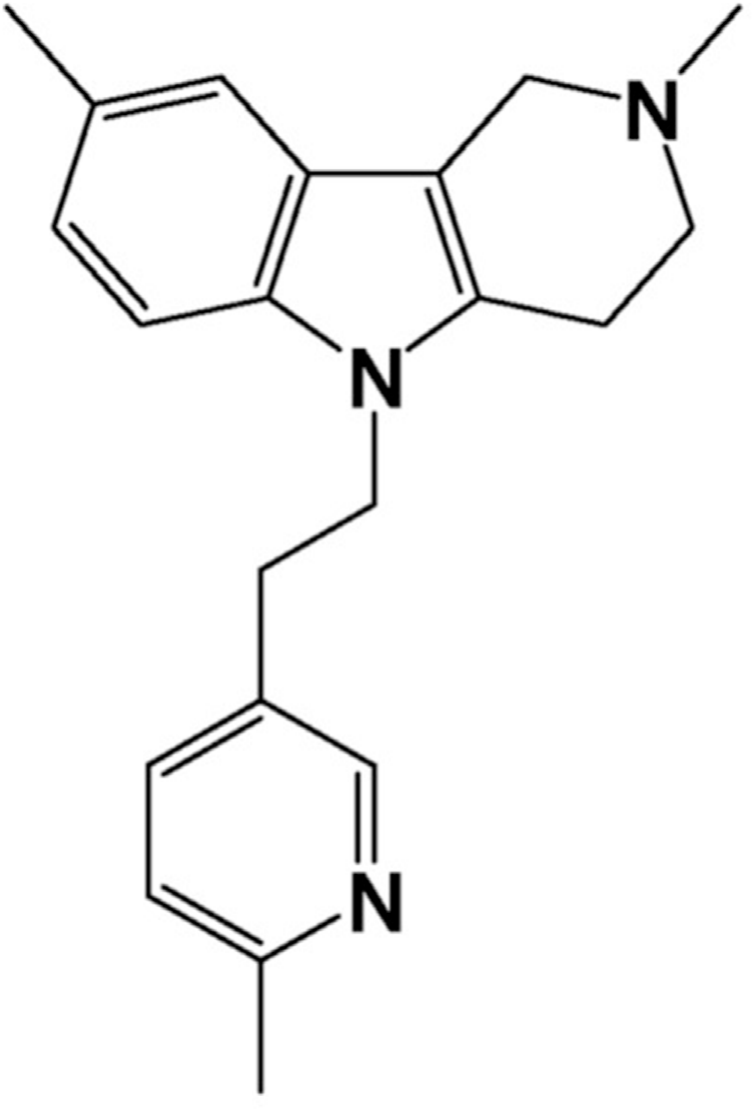
Latrepirdine structural formula.

Latrepirdine (Dimebon^®^, dimebolin or 2,3,4,5-tetrahydro-2,8-dimethyl-5- (2–6 (6-methyl-3-pyridyl) ethyl) -1H-pyrido (4.3-b) indole molecule (Figure 1), was discovered in 1973 (Kost et al., 1973) and approved for therapeutic application in Russia in 1983 as a non-selective antihistamine for allergy treatment but was gradually discontinued due to its low selectivity and efficiency. However, interest in latrepirdine resurged in early 2000 s due to its possible application as a neuroprotectant in pathologies such as Alzheimer’s disease (AD), and several clinical trials have been carried out in order to evaluate its efficacy. Cognition enhancing properties were first demonstrated in a small group of AD patients in an 8-week, open-label pilot study (Shadurskaia et al., 1986) followed by a large placebo-controlled phase II trial indicating substantial therapeutic benefit over placebo not only for cognitive symptoms and for activities of daily living, but also for neuropsychiatric (mainly affective) symptoms (Doody et al., 2008). However, the consecutive phase III multi-center, double-blind, placebo-controlled trials in AD patients failed to show positive effects of latrepirdine as a monotherapy compared with placebo (Jones, 2010). These negative data led to discontinuation of the further clinical development programs of latrepirdine. Given these contradictory findings and lack of clear understanding of the cause of discrepancies between different trials, we hypothesised that latrepirdine bioavailability and nootropic activity may be a function of its physicochemical characteristics, mainly polymorphism. The phenomenon of polymorphism is quite common among organic molecules, and many drugs can crystallize into different polymorphic forms with unique thermodynamic and physicochemical properties, such as energy, melting point, density, stability, and in particular solubility and dissolution rate, that may have a direct impact on pharmacokinetic and pharmacological properties of a drug substance. Several polymorphic forms of latrepirdine dihydrochloride, including crystalline anhydrous form A, form B hemi-hydrate, form C monohydrate, form D dihydrate, form F trihydrate, and amorphous form have been reported (WO 2009/111,540) previously. In addition, we recently reported a new crystalline form E of latrepirdine (WO 2022/160,066). In this communication we would like to report results of comparative study of bioavailability and nootropic activity of six crystalline polymorphs of latrepirdine.

## 2 Materials and methods

### 2.1 Pharmacokinetics

#### 2.1.1 Animals (pharmacokinetics)

177 mature specific pathogen free (SPF) male SD rats, 7–10 weeks old were obtained from the Pushchino nursery of laboratory animals (Pushchino, Russia). All procedures and manipulations with animals were approved by the Committee for Control over Care and Use of Laboratory Animals of BIBCh RAS (IACUC) (protocol number 809/21 from 10.08.2021) and were carried out in accordance with the EU Directive 2010/63/EU. After receiving from the nursery, the animals underwent adaptation within 7 days. During this period, the animals were monitored for signs of deviation in health status.Animals without signs of health deviations were selected for the experiment (clinical examination was done). Animals were divided into groups using the principle of randomization, using body weight as a criterion, so that the average body weight of animals by the first day of administration did not differ statistically between groups.Each animal was assigned an individual number, according to which the animal was marked with a puncture of the auricle. The cell label indicated the group, animal number, label, study code, head full name, IACUC protocol number, group color code. During the study, the animals were kept under controlled environmental conditions in a barrier zone with a “clean” and “dirty” corridor system with controlled environmental conditions (temperature 20°C–24°C, relative humidity 30%–55%, 12-h light cycle). 08:00–20:00c“day”, 20:00–08:00—“night”, 10-fold change in air volume in the room per hour). SNIFF RI/M-H V1534-30 complete granular rodent food was autoclaved and fed *ad libitum*.

#### 2.1.2 Drugs (pharmacokinetics)

Polymorphs A, B, C, D, E, and F were prepared from latrepirdinedihydrochloride (JV “Organica”) as described in Patent WO 2009/111540. New polymorph E was prepared from latrepirdine free base (JV “Organica”) as described in Patent WO 2022/160066.

#### 2.1.3 Pharmacokinetic study design (pharmacokinetics)

The preparations of six polymorphs of latrepirdine as a suspension in corn oil were orally administered to the animals for seven consecutive days, once daily at a dose of 10 mg/kg/5 mL. On the seventh day of administration, the animals were euthanized by anesthesia and subsequent exsanguination. According to the design of experiment (see Table 1), the animals were euthanized at different time points—15 min, 30 min, 60 min and 120 min after administration on the seventh day.

**TABLE 1:** Groups and doses (pharmacokinetics).

Group number	Subgroup number	Time point, minutes after the last injection on the 7th day of administration	Animal numbers	Drug, dose, volume, rout of administration, scheme
1	1	30	1-7	Corn oil, 5 mL/kg, ip for 7 days
2	2	120	8-14	Polymoiph A, 10 mg/kg, 5 mL/kg, po for 7 days
	3	60	15-21	
	4	30	22-28	
	5	15	29-35	
3	6	120	36-42	Polymoiph B, 10 mg/kg, 5 mL/kg, po for 7 days
	7	60	43-47,50*, 176,177	
	8	30	50-56	
	9	15	57-63	
4	10	120	64-70	Polymoiph C, 10 mg/kg, 5 mL/kg, po for 7 days
	11	60	71-77	
	12	30	78-84	
	13	15	85-91	
5	14	120	92-98	Polymoiph D, 10 mg/kg, 5 m/Lkg, po for 7 days
	15	60	99-105	
	16	30	106-112	
	17	15	113-119	
6	18	120	120-126	Polymoiph E, 10 mg/kg, 5 mL/kg, po for 7 days
	19	60	127-133	
	20	30	134-140	
	21	15	141-147	
7	22	120	148-154	Polymoiph F, 10 mg/kg, 5 mL/kg, po for 7 days
	23	60	155-161	
	24	30	162-168	
	25	15	169-175	

po – *per os* (orally by gavage)

* - in animals 48 and 49, by mistake, samples were taken 110 minutes after injection (instead of 60 minutes), for this reason, animals 176 and 177 were added to group 3, subgroup 7, which were injected with Polymorph B2 for 7 days, and then blood and brain samples were taken at the 60th minute after the last injection.

#### 2.1.4 Latrepirdine preparations (pharmacokinetics)

Individual administration doses of respective latrepirdine polymorph were prepared as follows: calculated amount of respective polymorph was weighed and mixed with corn oil in a volume that is equivalent to 5 mL/kg of an animal. Using an automatic pipette, the resulting suspension was transferred into a vial continuously stirred with magnetic stirrer and the suspension was dosed to animals as required. The preparation of doses for administration was carried out immediately before administration (no more than 3 h before administration) on the days of administration. Labeled doses ready for administration in vials were transferred to the animal holding area immediately before administration.

#### 2.1.5 Drugs administration (pharmacokinetics)

The preparations containing latrepirdine polymorphs were administered by gavage at a dose of 10 mg/kg/5 mL daily once a day for 7 days. The body weight of the animal was used to calculate the individual administration volume of 10 mg/kg for each latrepirdine polimorph. The administration of latrepirdine preparations was carried out according to the schedule of procedures at the same time of day (09:00–13:00). Vials with a suspension of preparations were kept on a magnetic stirrer when dosing.

#### 2.1.6 Sample collection (pharmacokinetics)

On the day of necropsy, blood was taken from anesthetized animals from the inferior vena cava, placed in a test tube with sodium citrate 1:9 (1 part sodium citrate: nine parts blood). Blood was centrifuged at 21°C, 1600 g, 15 min, blood serum was collected (at least 600 µL), placed in marked Eppendorf tubes and frozen at −70°C and stored at −70°C until being analyzed. After blood sampling from the inferior vena cava, the brain was removed from the cranium, divided into two hemispheres in the sagittal direction, one of the hemispheres was frozen in liquid nitrogen and placed in labeled containers, which were stored in a freezer at −70°C until analyzed.

#### 2.1.7 Sample analysis (pharmacokinetics)

The concentration of latrepirdine in the liquid extracts of blood serum and brain tissue of rats was analyzed using liquid chromatography-mass spectrometer “LCMS-8050” equipped withg LC-20 Prominence chromatograph and SIL 20 Axrautosampler (Shimadzu, Japan) as described elsewhere (Nirogi et al., 2009). Polymorph Latrepirdine A substance, series No. 10121, was used as the analytical standard. Liquid extraction was used to extract the analyte. For quantitative determination, the HPLC-MS method (high performance liquid chromatography-mass spectrometry) was used with electrospray ionization and ion registration in the MRM mode (registration of multiple transitions), which allows achieving high sensitivity and selectivity of the technique. Quantitative determination of the substance content in blood serum and brain tissue was carried out by the method of an external standard or absolute calibration. Analyte identification is carried out by retention parameters and mass spectrometric data when comparing experimental data obtained for calibration and test solutions.

#### 2.1.8 Extraction from blood serum (pharmacokinetics)

To 300 μL of blood serum was added 50 μL of 0.1 M NaOH solution, stirred, then 1.2 mL of methyl tert-butyl ether: hexane (80:20) was added. Thoroughly mixed on an orbital shaker for 10 min, then the sample was centrifuged at 13,000 rpm for 5 min. The top layer was taken and evaporated to dryness. To the dry residue was added 0.300 mL of a 30% aqueous solution of acetonitrile, thoroughly mixed. The solution was transferred to an autosampler vial.

#### 2.1.9 Extraction from brain homogenate (pharmacokinetics)

A sample of brain tissue (300 mg) was homogenized using an ultrasonic homogenizer. To the homogenate was added 50 μL of 0.1 M NaOH solution, stirred. Then 1.2 mL of methyl tert-butyl ether was added, thoroughly mixed on an orbital shaker for 15 min. The sample was centrifuged at 13,000 rpm for 5 min. The top layer was taken and evaporated to dryness. To the dry residue was added 0.3 mL of a 30% aqueous solution of acetonitrile, thoroughly mixed. The solution was transferred to an autosampler vial.Chromatography conditions are shown in Table 2.

**TABLE 2:** Chromatography conditions.

Column	Kinetex С18 5 µm (100 × 2.1) mm
Mobile Phase	Component A- 0.1% aqueous solution of formic acid
	Component B- 0.1% solution of formic acid in acetonitrile
Chromatographic elution mode	Isocratic, the ratio of components A and B is 70:30
Eluent flow rate	0.4 mL/min
Column oven temperature	40°С
Administration volume	10 µL
Retention time of latepirdine	0.55 min
Analysis time	0.7 min
Column washing and conditioning mode	0.7 min—30%B; 0.8–1.90 min—85% B; 2.00–4.00 min—30%B

#### 2.1.10 Calibration (pharmacokinetics)

A standard solution of a standard sample (RS, latrepirdine hydrochloride) was prepared as follows: a standard sample of 5 mg was placed in a 2 mL microtube, dissolved in 1 mL of a 30% aqueous solution of acetonitrile with 0.1% formic acid (30 ACN +0.1FA), thoroughly mixed (hereinafter using a shaker for 1 min). 200 μL of standard solution was added to a microtube, 800 µL of 30 ACN + 0.1FA was added to obtain a 1 mg/mL solution. By successive dilution, solutions were obtained with a concentration of 500; 100; fifty; 10 and 5 μg/mL. To establish the calibration characteristics, samples for calibration were prepared by adding the appropriate working solutions (WS) of latrepirdine to intact rat blood blood serum to obtain concentrations of latrepirdine 1, 5, 15, 30, 60, and 75 ng/mL and the introduction of the appropriate working solutions (WS) of latrepirdine in the homogenizate of intact rat brain tissue to obtain concentrations of 3, 5, 15, 30, 60, and 100 ng per 300 mg of brain tissue. The prepared solutions were labeled and used to construct calibration curves. Chromatograms were registered and processed using the LC Solution software (Shimadzu, Japan) for quantitative processing of the analytical results.

#### 2.1.11 Statistical analysis (pharmacokinetics)

Analytical data for concentrations of latrepirdine in blood serum and in the brain tissue were processed using the Statistica 7.1 program. Descriptive statistic approach was applied: for the concentrations of latrepirdine in blood serum and in the brain tissue, the mean value and standard deviation were calculated, which are presented in the tables below. Two-way analysis of variance factorial ANOVA (ANOVA 2) was used to establish statistically significant differences associated with the factors “time” and “drug”. To determine statistically significant differences in concentration between administered polymorphs at different time points, one-way ANOVA (ANOVA 1) and Kruskal–Wallis ANOVA analysis were used. Differences were determined at the 5% significance level (*p* ≤ 0.05).

### 2.2 Study of cognitive-enhancing nootropic effect of latrepirdine polymorphs in rats

#### 2.2.1 Animals (cognitive-enhancing nootropic effect)

The study of nootropic activity was carried out on 64 laboratory Wistar rats of both sexes (32males and 32 females), 6–8 weeks old, weighing 200–250 g. The animals were obtained from the Rappolovo nursery of the National Research Center Kurchatov Institute, Russia. All procedures and manipulations with animals were approved by the Committee for Control over Care and Use of Laboratory Animals of Federal Research Center of Kazan Scientific Center, Russian Academy of Sciences, Russia (IACUC) (protocol number two from 02.07.2022) and were carried out in accordance with the EU Directive 2010/63/EU. Laboratory animals were kept in cages for at least 3 days prior to the start of the research. During this period, the animals were monitored daily for clinical condition by visual inspection. Clinically healthy animals were transferred to the experiment. During the period of adaptation and experiment, the animals were kept in standard transparent polycarbonate cages in groups of four individuals, on the bedding; cages are covered with steel lattice covers with aft recess. The animals were given drinking water *ad libitum*. The animals were kept under controlled environmental conditions (temperature from 19°C to 25°C and relative humidity from 30% to 70%) and light mode (day/night) 12/12 h.

#### 2.2.2 Scopolamine use for modeling of Alzheimer’s disease (cognitive-enhancing nootropic effect)

The administration of scopolamine, an antagonist of muscarinic acetylcholine receptors, causes a temporary blockade of receptors, impaired cognitive functions and cerebral metabolism, which is considered to be a reliable model of AD (Buccafusco, 2009). Scopolamine aqueous solution was prepared by dissolving weighed sample in water for injection. A solution of scopolamine was injected intraperitoneally at a dose of 1.5 mg/kg/5 mL once 30 min before testing of the passive avoidancereflex (only on the first day - the day of training).

#### 2.2.3 Latrepirdine preparation and administration (cognitive-enhancing nootropic effect)

Individual administration doses of respective latrepirdine polymorph were prepared as follows: calculated amount of respective polymorph was weighed and mixed with corn oil in a volume that is equivalent to 5 mL/kg of an animal. The mixture was triturated with aid of porcelain mortar and pestle until a homogeneous suspension is obtained. The resulted suspension was sonicated for 10 min at ambient temperature the prepared doses were administered within 3 h on the days of experiment to avoid polymorph transitions. Suspensions of the polymorphs were administered orally (*per os*—po) using by gavageat a dose of 10 mg/kg/5 mL daily once a day for 9 days.

#### 2.2.4 scopolamine-induced memory impairment in rats using passive avoidance (PA) conditioning to evaluate—Study design (cognitive-enhancing nootropic effect)

The administration of scopolamine, an antagonist of muscarinic acetylcholine receptors, causes a temporary blockade of these receptors that impaired cognitive functions (Buccafusco, 2009).

For 8 days, animals received oraly latrepirdine polymorphs according to group affiliation (Table 3). Polymorph preparations of latrepirdine at a dose of 10 mg/kg were administered to experimental animal groups (groups 3–8) once a day for 7 days. The groups 1 and 2 were administered once a day with an equivalent amount of corn oil.

**TABLE 3:** Groups and doses (cognitive-enhancing nootropic effect).

Group number	Damage agent dose, volume, rout of administration, scheme	Drug, dose, volume, rout of administration, scheme	Animal number	
			Males	Females
1	-	Corn oil, 5 mL/kg, ip for 9 days	1–4	5–8
2	Scopolamine solution, 1.5 mg/kg, 5 mLkg, ip 30 min before training	Corn oil, 5 mL/kg, po for 9 days	9–12	13–16
3		Polymorph A, 10 mg/kg, 5 mL/kg, po for 9 days	17–20	21–24
4		Polymorph B, 10 mg/kg, 5 mL/kg, po for 9 days	25–28	29–32
5		Polymorph C, 10 mg/kg, 5 mL/kg, po for 9 days	33–36	37–40
6		Polymorph D, 10 mg/kg, 5 m/Lkg, po for 9 days	41–44	45–48
7		Polymorph E, 10 mg/kg, 5 mL/kg, po for 9 days	49–52	53–56
8		Polymorph F, 10 mg/kg, 5 mL/kg, po for 9 days	57–60	61–64

Po—*per os* (orally by gavage).

To study cognitive-enhancing nootropic effect of the polymorphs the passive avoidance (PA) test was performed as described by Burwell et al. (Burwell et al., 2004). The PA apparatus (Neurobotics, Moscow, Russia) consisting of two compartments (light and dark chambers) separated by sliding guillotine door was used. PA consisted of an acquisition trial on the first (training) day and a retention trial 24 h later. On the seventh day of corn oil or the test polymorph administration, the animals were placed for 5 min in the PA unit to familiarize themselves with the space in order to avoid the stress caused by the new environment. The next day (eighth day) the training phase of PA test was started. 60 min before the start of training, corn oil or the test polymorph was administered, as per the group design. The experimental groups (2–8) were injected intraperitoneally with scopolamine at a dose of 1.5 mg/kg 30 min before training. During the training phase of PA test each rat was placed in the lighted chamber. After 10 s, the door was opened, and latency to enter the dark chamber was recorded. An animal was considered to have entered the dark chamber when all fours paws were beyond the guillotine door. After entry, the door was closed. After 2 s had elapsed, a foot shock stimulus was delivered. The shock level and duration were set at 1 mA for 2 s. The rat was immediately removed back to its home cage. Twenty-4 hours later (ninth day), a retention test consisted of placing the rat back into the lighted chamber, waiting 10 s, opening the door, and the latent period (LP) of the first entry into the dark chamber was recorded. If a rat did not enter the chamber in 180 s, the trial was terminated, and the rat was removed to the home cage.

The most effective polymorph was determined.

#### 2.2.5 Statistical analysis (cognitive-enhancing nootropic effect)

Statistical processing of the results of a comparative study of the cognitive-enhancing activity of polymorphs was performed using the Statistics 7.1 software. Pairwise comparison using the Mann-Whitney *U*-test was used to identify differences between each polymorph with control and with scopolamine, as well as differences between polymorphs. The significance level was determined at *p* ≤ 0.05.

## 3 Results

### 3.1 Pharmacokinetics

Pharmacokinetics study of six latrepirdine polymorphs (A, B, C, D, E, F) after 7 days of administration with blood serum and brain sampling on the seventh day of administration at 15 min, 30 min, 60 min and 120 min after the last administration and subsequent analysis of the concentration of latrepirdine revealed the following data presented below (Table 4).

**TABLE 4:** The concentration of Polymorphs in blood serum, ng/ml, average values.

	Polymorph А	Polymorph B	Polymorph C	Polymorph D	Polymorph E	Polymorph F
15 min	2.0 ± 2.1	5.9 ± 1.7	7.4 ± 7.1	8.7 ± 7.7	12.8 ± 10.5	4.8 ± 2.2
30 min	7.0 ± 11.5	5.6 ± 2.1	6.8 ± 4.3	12.9 ± 12.1	10.0 ± 7.0	6.3 ± 4.2
60 min	3.9 ± 2.1	7.4 ± 2.7	6.7 ± 2.4	11.6 ± 2.9	9.7 ± 2.2	9.4 ± 4.5
120 min	2.0 ± 0.6	1.0 ± 0.7	7.0 ± 2.4	12.2 ± 9.3	10.4 ± 4.8	3.6 ± 0.8

Data are presented as MEAN ± SD.

**FIGURE 2:**
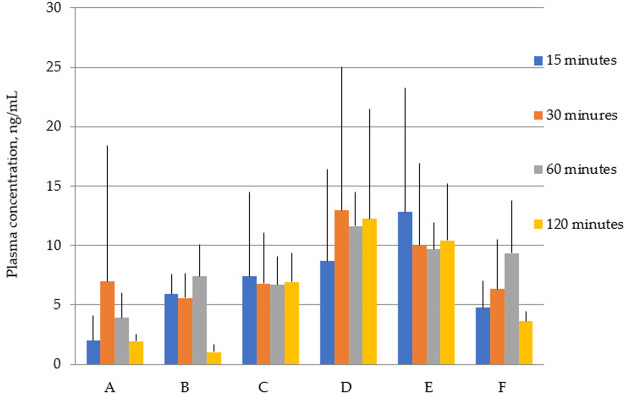
Concentration of latrepirdine for polymorphs **(A–F)** in blood serum samples. Data are presented as mean values for each polymorph at a specific time point + standard deviation.

**FIGURE 3:**
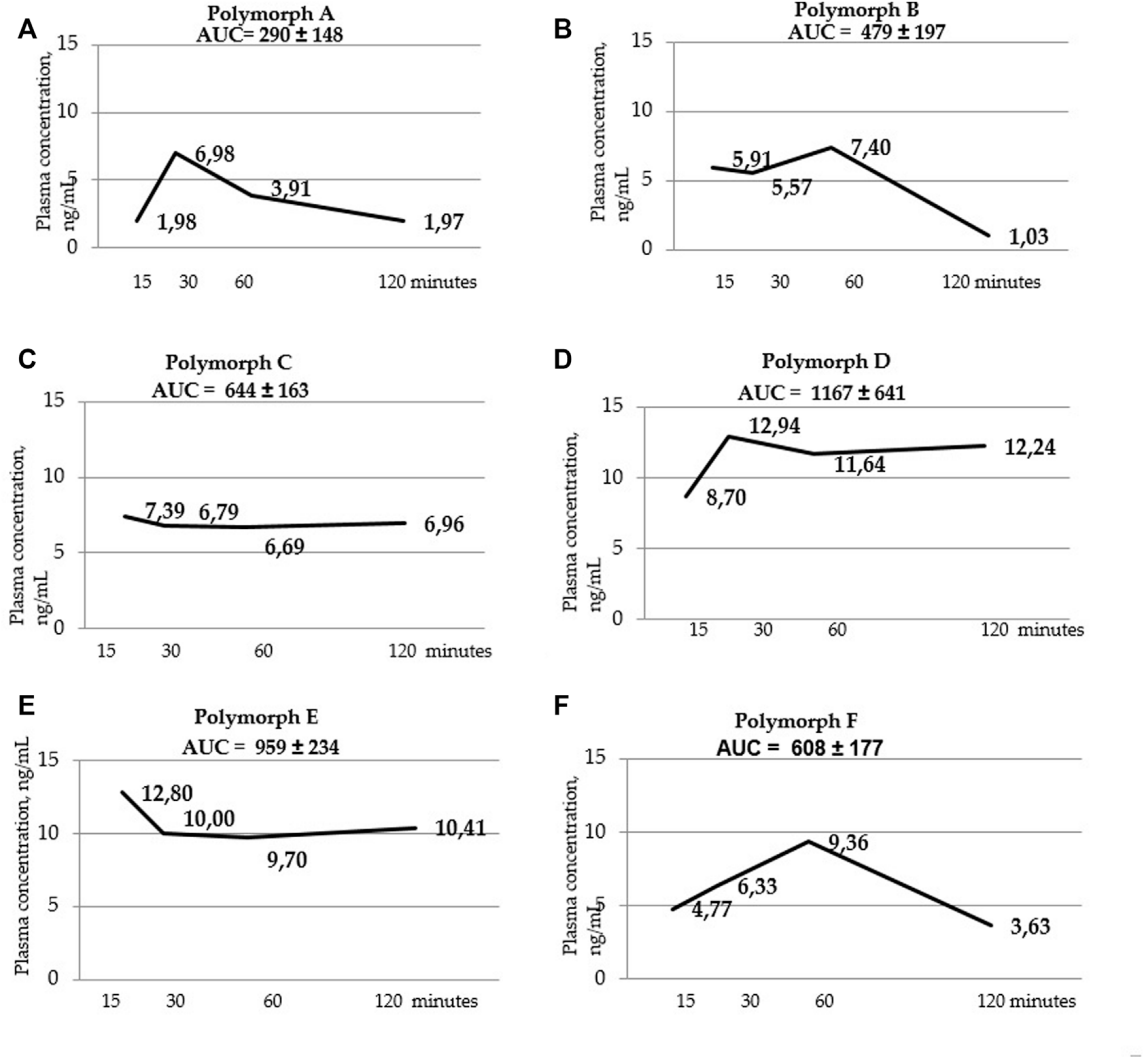
Blood serum concentration-time curves for polymorphs **(A–F)**. AUC values are listed at the top of the graphs. AUC values were calculated using the trapezoid method for each individual animal, then the mean value and standard deviation of the AUC value for each polymorph were calculated.

Figure 2 shows that the total concentrations of latrepirdine after oral administration of polymorphs D and E are greater than for the rest of the polymorphs. This is also confirmed by the AUC values (Table 5; Figure 3).

**TABLE 5:** Blood serum AUC values calculated by the trapezoidal method.

Polymorph	AUC
А	290 ± 148
B	479 ± 197
C	644 ± 163
D	1,167 ± 641^a^
E	959 ± 234^a^
F	608 ± 177

Data are presented as group means ± standard deviation.

^a^ p ≤ 0.05 relative to the AUC, value for polymorph A according to the Kruskal–Wallis test (see also Figure 2).

Below are the blood serum concentration *versus* time curves and AUC values calculated using the trapezoid method (Figure 3 a, b, c, d, t, f).

**FIGURE 4:**
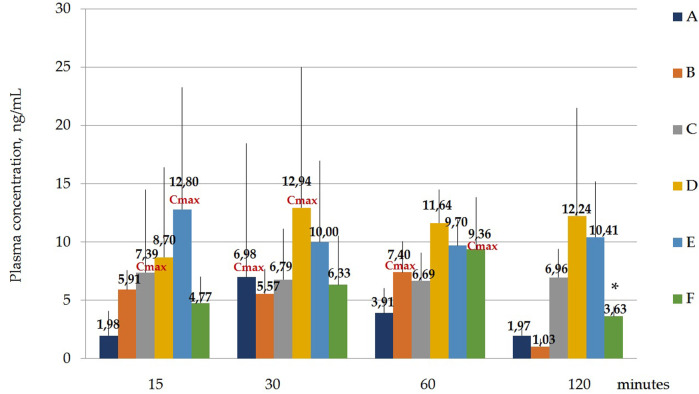
Concentration of laterpirdine for polymorphs **(A–F)** in blood serum samples. Data are presented as means of all polymorphs at each time point + standard deviation. The graph shows the values of the maximum concentration for each polymorph (Cmax). Blood Tmax is the time point at which Cmax is observed. *—*p* ≤ 0.05 relative to the “60 min” time point for each Polymorph according to the Kruskal–Wallis test.

Figure 4 and Table 6 below show the maximum blood serum concentrations (Cmax) and the time at which the maximum concentration (Tmax) is observed for all polymorphs.

**TABLE 6:** Cmax and Tmax values in blood serum.

Polymorph	А	B	C	D	E	F
C max, ng/ml	6.98	7.40	7.39	12.94	12.8	9.36*^a^*
T max, min	30	60	15	30	15	60

^a^ - p ≤ 0.05 relative to the “60 min” time point for each Polymorph according to the Kruskal–Wallis test.

**FIGURE 5:**
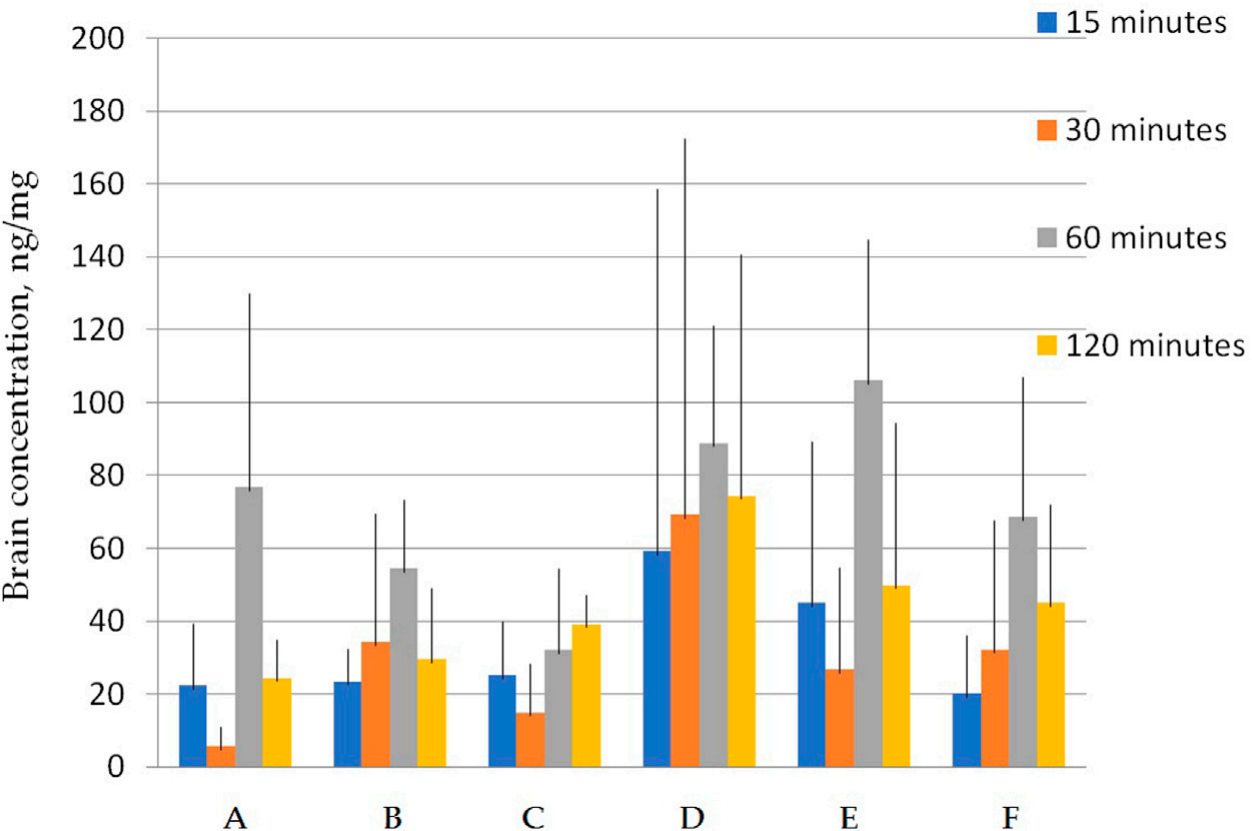
Concentration of latrepirdine for polymorphs **(A–F)** in brain tissue samples. Data are presented as mean values for each polymorph at a specific time point + standard deviation.

Hence, it was shown that the concentrations of latreperdine for each of six polymorphs at different time points do not statistically differ from each other, including Tmax, with the exception of the F polymorph, whose Cmax attributable to the time point “60 min” is significantly higher than the concentration at the point “120 min”, *p* = 0,007,786 (Table 7; Figure 5).

**TABLE 7:** Concentration of latrepirdine in brain tissues, ng/mg—individual data and mean values.

	Polymorph А	Polymorph B	Polymorph C	Polymorph D	Polymorph E	Polymorph F
15 min	22.4 ± 16.8	23.5 ± 8.7	25.1 ± 14.6	59.2 ± 99.4	45.1 ± 44.1	20.2 ± 15.7
30 min	5.6 ± 5.4	34.2 ± 35.1	15.0 ± 13.3	69.1 ± 103.0	26.8 ± 27.9	32.2 ± 35.5
60 min	76.8 ± 53.0	54.4 ± 18.8	32.1 ± 22.1	88.9 ± 32.1	106.0 ± 38.6	68.5 ± 38.3
120 min	24.4 ± 10.4	29.7 ± 19.3	39.1 ± 7.8	74.4 ± 66.2	49.8 ± 44.4	45.1 ± 26.8

Data are presented as MEAN ± SD.

- concentration was below the detection threshold.

**FIGURE 6:**
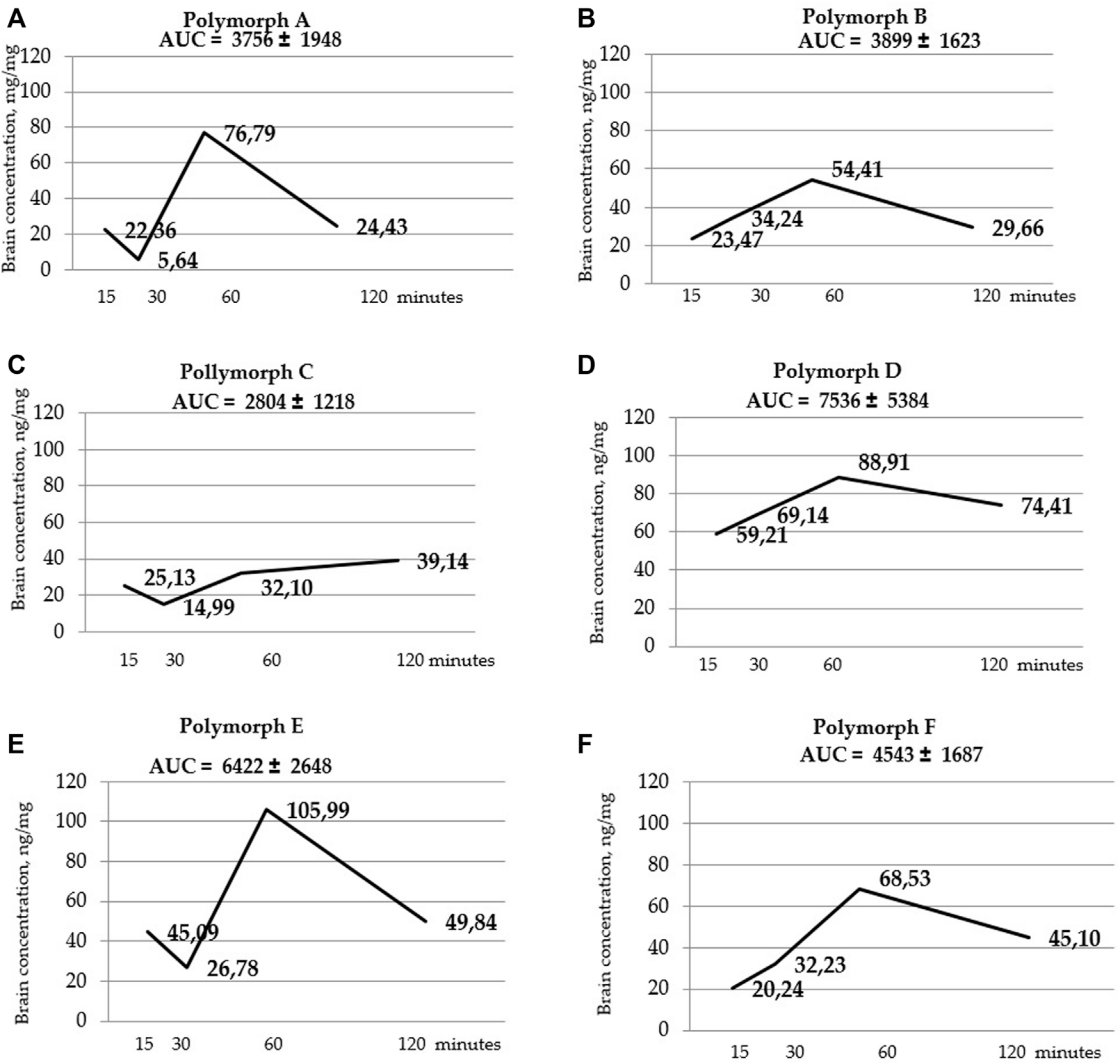
Curves of latrepirdine concentration in brainversus time for polymorphs **(A–F)**. AUC values are listed at the top of the graphs under the name of the polymorph. AUC values are calculated using the trapezoidal method.

Figure 5 shows that the total concentrations of D and E polymorphs are greater than those of the other polymorphs. This is also confirmed by the AUC values (Table 7; Figure 6).

**FIGURE 7:**
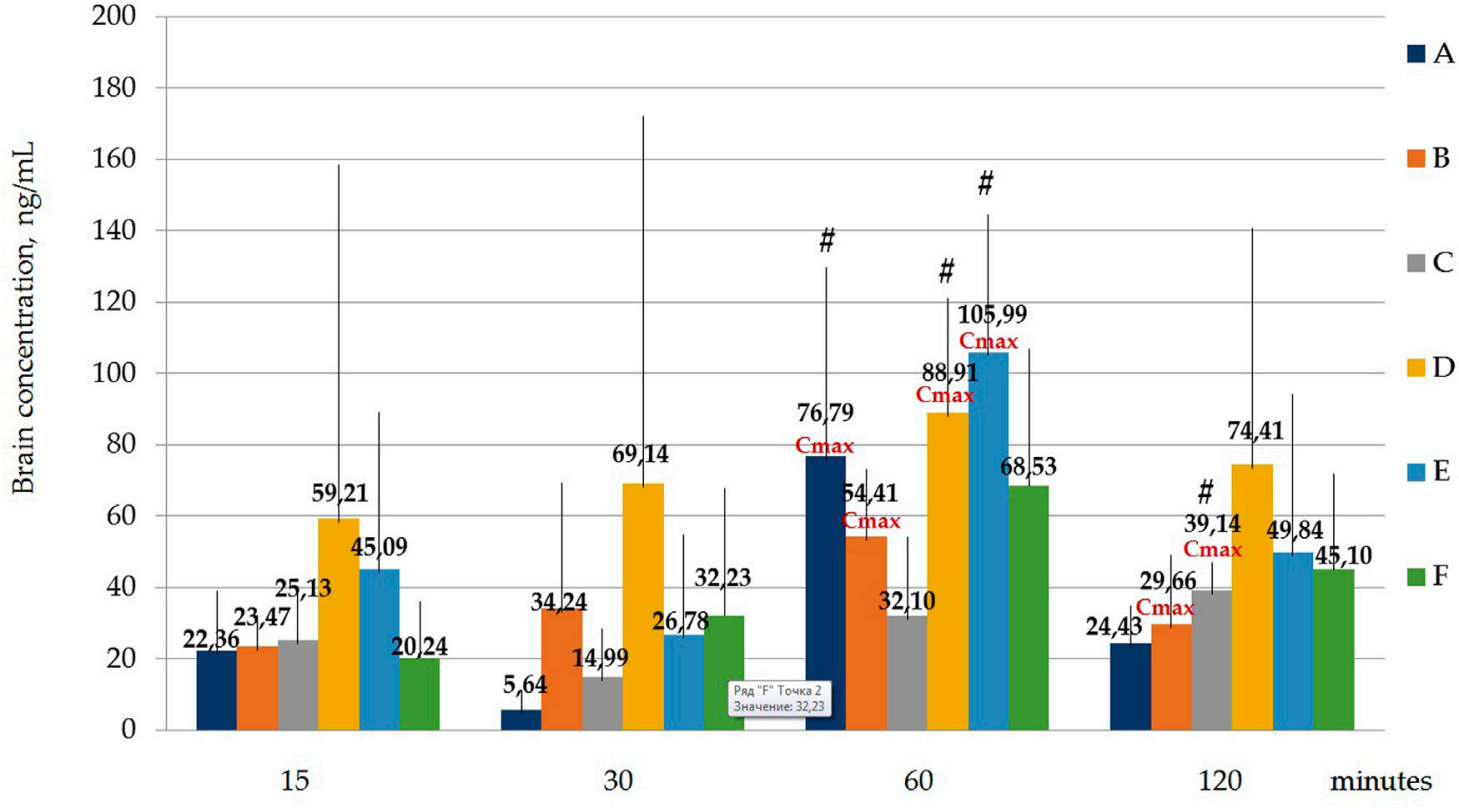
Concentration of latrepirdine for polymorphs **(A-F)** in brain samples. Data are presented as means of all polymorphs at each time point +standard deviation. #—*p* ≤ 0.05 relative to the “30 min” time point for each Polymorph according to the Kruskal–Wallis test.

Figure 7 and Table 9 Below shows the values of the maximum concentration for each polymorph (Cmax). Tmax is the time point at which Cmax is observed.

It was shown that the Cmax for polymorph A observed at the time point “60 min” was significantly higher than the concentration at the time point “30 min”, *p* = 0.001394; for polymorph B, Cmax observed at the time point “60 min” does not significantly differ from the concentrations at other time points, but the concentration at the time point “30 min” significantly differs from the concentration at the time point “120 min”, *p* = 0.036263; Cmax for polymorph D observed at the time point “60 min” is significantly higher than the concentration at the time point “30 min”, *p* = 0.010395; Cmax for polymorph E observed at the time point “60 min” is significantly higher than the concentration at the time point “30 min”, *p* = 0.010395.

Using a two-way ANOVA two analysis, it was shown that in blood serum there is an obvious dependence between the administered latrepirdine polymorphic form and its concentration. Post hoc analysis revealed that the oral administration of polymorphs D and E led to higher concentration of latrepirdine in blood at all of the time points in comparison with other Polymorphs. This is supported by the AUC values calculated for each polymorph (the value characterizing the bioavailability), whereby polymorphs D and E have the highest values while polymorph A the lowest one.

A one-way analysis performed to identify differences in blood serum concentrations between Polymorphs at each time point showed that the largest differences at the “15 min” point - the concentration of latrepirdine for polymorph E is statistically significantly higher than the concentrations for polymorphs A, C and F. At the “30 min” point, there were no differences between the concentrations of latrepirdine for all polymorphs. At the “60 min” point, the concentration of latrepirdine for polymorph D was statistically higher than for polymorph A. At the point “120 min” the concentration of latrepirdine for polymorphs A, B and F decreased relative to the point “60 min”, and its concentration was significantly lower than the concentration for Polymorphs C, D and E (*p* ≤ 0.05), while the concentrations for Polymorphs C, D and E did not decrease relative to the concentrations for these polymorphs at the 60 min point, but reached a plateau. Peak blood serum concentrations of latrepirdine for polymorphs were observed at different time points.

Summarizing the data on the pharmacokinetic distribution of latrepirdine in the blood serum of male SD rats for the six studied polymorphs, we can conclude that bioavailability of polymorphs D and E differs significantly from that of other polymorphs. These polymorphs have the highest AUC values, indicating their higher blood serum bioavailability compared to other polymorphs.

Statistical calculation of the data obtained for brain samples using two-way ANOVA two show that the distribution of latrepirdine in brain tissues also depends on the polymorphic form and time. Post hoc analysis showed that the highest levels of latrepirdine were observed for polymorphs D and E, which is confirmed by the highest AUC values for these polymorphs (Figure 6; Table 8). Regarding the “time” factor, at the time point “60 min”, the concentrations differed from the rest of the time points. In brain tissues, the maximum concentration Cmax for all polymorphs except polymorph C was observed at 60 min, while for polymorph C it was at 120 min (Figure 7; Table 9). For all polymorphs, latrepirdine concentrations in brain decreased at 120 min, except for polymorph C, which plateaued at 120 min.

**TABLE 8:** AUC values for brain tissue samples calculated by the trapezoid method.

Polymorph	AUC
А	3,756 ± 1948
B	3,899 ± 1,623
C	2,804 ± 1,218
D	7,536 ± 5,384
E	6,422 ± 2,648^a^
F	4,543 ± 1,687

Data are presented as group means ± standard deviation.

^a^ p ≤ 0.05 relative to the AUC, value for polymorph C according to the Kruskal–Wallis test (see also Figure 6).

**TABLE 9:** C max and T max values for brain tissue samples.

Polymorph	А	B	C	D	E	F
Cmax, ng/mg	76.8 ± 53.0*^a^*	54.4 ± 18.8	39.1 ± 7.8*^a^*	88.9 ± 32.1*^a^*	106.0 ± 38.6*^a^*	68.5 ± 38.3
Tmax, min	60	60	120	60	60	60

Data are presented as group means ± standard deviation.

^a^ p ≤ 0.05 relative to the “30 min” time point for each Polymorph according to the Kruskal–Wallis test.

### 3.2 Study of cognitive-enhancing nootropic effect of latrepirdine polymorphs in rats

The nootropic efficacy of six polymorphs (A-F) of latrepirdine at a dose of 10 mg/kg was compared. The dose was chosen based on the fact that polymorph E had a statistically significant nootropic effect at a dose of 10 mg/kg. Moreover, a further increase in the dose to 20 mg/kg does not lead to a statistically significant increase in the effectiveness of therapy.

#### 3.2.1 Passive avoidance testing (study of cognitive-enhancing nootropic effect of latrepirdine polymorphs in rats)

Mean LP values are presented in Table 10.

**TABLE 10:** Latent period (LP) of transition to the dark chamber in PA test 24 h after training.

Group number	Drugs	Mean LP ± SEM, sec	Differences with vehicle (group 1), *p*-value	Differences with respect to scopolamine (group 2), *p*-value
1	Vehicle - Corn oil	177 ± 3		
2	Scopolamine + Corn oil	44.87 ± 13.3	p = 0,000,778	
3	Scopolamine + Polymorph A	44.25 ± 13.25	p = 0.000778	*p* = 0.916
4	Scopolamine + Polymorph B	68.125 ± 20.33	p = 0.003876	*p* = 0.4948
5	Scopolamine + Polymorph C	103.75 ± 22.92	p = 0.015715	*p* = 0.0928
6	Scopolamine + Polymorph D	49.63 ± 14.71	p = 0.000778	*p* = 0.95812
7	Scopolamine + Polymorph E	159.13 ± 8.65	*p* = 0.14148	p = 0.000778
8	Scopolamine + Polymorph F	101.875 ± 19.4	p = 0.015715	*p* = 0.058708

Data are presented as Mean value in seconds ± SEM., Dose for all polymorphs was 10 mg/kg. Differences were considered statistically significant at p < 0.05 (Mann-Whitney U-test), highlighted in bold.

Analysis of the presented data allows us to say that only one of the latrepirdine forms studied, namely, polymorph E, has a statistically significant increase in LP at a dose of 10 mg/kg compared with the group of animals treated with scopolamine, but not receiving latrepirdine. It is important to note that LP values of the control group of animals (group 1) did not differ from the group that received polymorph E.Also, when polymorphs and scopolamine were compared in pairs, only polymorph E differed from scopolamine (Table 10).

Pairwise comparison of polymorphs with each other revealed that polymorph E statistically differs from all other polymorphs, exceptpolymorph C, in the direction of increasing latency into the dark chamber. The remaining polymorphs do not differ from each other (Table 11).

**TABLE 11:** Analysis of statistically significant differences between the effects of all polymorphs.

No.	Compared pairs of polymorphs	Differences between polymorphs, *p*-value
1	А + В	р = 0.4308
2	А + С	р = 0.127
3	A+ D	р = 0.7527
4	A+ E	р = 0.00071
5	A+ F	р = 0.0.0587
6	B+ C	р = 0.27
7	B+ D	р = 0.5995
8	B+ E	р = 0.00632
9	B+ F	р = 0.2274
10	C + D	р = 0.11518
11	C + E	р = 0.092
12	C + F	р = 1.000
13	D + E	р =0.00113
14	D + F	р = 0.08312
15	E + F	р = 0.03569

Note - *p* values ≤ 0.05 are in bold.

Thus, it can be concluded that polymorph E is most effective in comparison with other polymorphs when testing long-term memory in the PA test.

## 4 Discussion

The dose of 10 mg/kg for all polymorphs was chosen based on previous studies. For example, in a study by Jun et al. studied the efficacy of latrepirdine (Dimebon^®^) in a rat model of Alzheimer’s disease after 4 months of intraperitoneal administration at a dose of 12 mg/kg. In this study, the administration of Dimebon^®^ improved the results of testing navigational memory in the Morris water maze (Wang et al., 2011).In another study, oral administration of Dimebon^®^ at a dose of 5 mg/kg 30 min before testing improved the memory of animals in a novel object recognition test (Giorgetti et al., 2010).

In our study of the pharmacokinetics of latrepirdine polymorphs, the bioavailability of all polymorphs in both plasma and brain was shown. Moreover, the concentrations in the brain were higher in comparison with the concentrations in plasma. The obtained data are consistent with the previous study (Wang et al., 2011), where the concentration of latrepirdine was measured after a course of oral administration.In this study, it was shown that the concentration in the brain is almost 4 times higher than the concentration in plasma. In our study, the concentration in the brain exceeded the concentration in plasma by about 5 times. We have also shown that different polymorphs have different bioavailability, characterized by AUC. That is, it can be argued that the crystalline form of latrepirdine affects the bioavailability of the drug.

To study the effectiveness of latrepirdine polymorphs against Alzheimer’s disease, we used a single injection of the cholinergic receptor blocker scopolamine. Scopolamine is used to study cognitive-enhancing drugs (Ebert and Kirch, 1998). An advantage of using scopolamine to study drugs with a putative effect on cognition is the reversibility of its damaging effect of transient amnesia (Bartus, 1978). The utility of using scopolamine as a model for Alzheimer’s is supported by the ability of central cholinesterase inhibitor drugs for AD therapy, such as rivastagmine, donepezil, and galantamine, to improve animal memory by reversing the effects of scopolamine (Bartus, 1978; Mewaldt and Ghoneim, 1978; Bejar et al., 1999; Misane and Ogren, 2003; Takahata et al., 2005; van der Staay and Bouger, 2005; de Bruin and Pouzet, 2006; Skalicka-Wozniak et al., 2018; Yadang et al., 2020). In AD, damage occurs to cholinergic neurons in specific parts of the brain, and presynaptic M2 receptors located on the cholinergic basal projection neurons of the forebrain are damaged (Aubert et al., 1992).

Latrepirdine belongs to a growing group of “old” drugs that are expected to have therapeutic activity different from that previously claimed (Peters et al., 2013). In our study on the scopolamine-induced D model, the latrepirdine polymorph E showed efficacy in terms of memory improvement in the passive avoidance reflex formation test. Our results confirm the previously found efficacy of latrepirdine shown in a number of studies. So on models of cholinergic deficiency caused by the use of neurotoxins AF64 (Lermontova, 2000) and a fragment of *β*-amylyloid Аβ25-35 (Lermontova, 2001). Chronic oral administration of latrepirdine for 5 months to transgenic mice with severe motor impairment improved the motor functions of animals (de Bruin and Pouzet, 2006). Also, a number of clinical studies have shown the effectiveness of latrepirdine in patients with asthma (Shadurskaia et al., 1986; Doody et al., 2008).

The mechanisms of neuroprotection of latrepirdine are diverse. For example, Egea et al. it was shown that in ischemic brain damage in rats, the administration of dimebon affected several links of the ischemic cascade, preventing neuronal death, namely, it reduced iNOS induction and ROS production, reduced mitochondrial depolarization, stabilizing their function, and also reduced glutamate-induced intracellular Ca_2+_ overload in hippocampal neurons, which reduces hyperstimulation of NMDA receptors, thus reducing excitotoxicity, which is one of the key death mechanisms of ischemic neurons (Egea et al., 2014). It has also been shown that dimebon reduces the pathological permeability of mitochondria in Alzheimer’s disease (Shevtsova et al., 2014).

Despite the fact that recent clinical studies of latrepirdine in relation to the effectiveness of AD therapy were not successful, a new crystalline form of this drug, namely, polymorph E, developed and studied by us, showed efficacy in relation to AD. This polymorph E also showed high bioavailability. Thus, the latrepirdine E polymorph is a promising drug for the treatment of AD and requires further study in this direction.

## 5 Conclusion

It has been shown that the greatest concentration of latrepirdine in the blood serum and brain tissue samples of SD rats after oral administration of the studied six polymorphs of latrepirdine were observed for polymorphs D and E. This is confirmed by the highest AUC values for these two polymorphs, which indicates their higher bioavailability in blood and brain tissues compared to other polymorphs.

With regards to the improvement of cognitive functions, the obtained results indicate that one of latrepirdine polymorphs studied, namely, polymorph E, at a dose of 10 mg/kg, shows a significant nootropic activity, restoring the disrupted conditioned passive avoidance reflex caused by injection of scopolamine in rats. Other polymorphs of latrepirdine at a dose of 10 mg/kg do not exhibit a statistically significant therapeutic effect. Thus, it can be concluded that the latrepirdine E polymorph has therapeutic activity against scopolamine-induced memory impairment, and this polymorph is the only polymorph of all studied that showed such activity.

## Data Availability

The original contributions presented in the study are included in the article/supplementary materials, further inquiries can be directed to the corresponding author.
